# Indirect estimation of reference intervals for thyroid parameters using advia centaur XP analyzer

**DOI:** 10.5937/jomb0-33543

**Published:** 2022-04-08

**Authors:** Bosa Mirjanić-Azarić, Neda Milinković, Natasa Bogavac-Stanojević, Sanja Avram, Tanja Stojaković-Jelisavac, Darja Stojanović

**Affiliations:** 1 University of Banja Luka, Faculty of Medicine, Banja Luka, Bosnia and Herzegovina; 2 University Clinical Centre of the Republic of Srpska, Institute of Laboratory Diagnostic, Banja Luka, Bosnia and Herzegovina; 3 University of Belgrade, Faculty of Pharmacy, Department of Medical Biochemistry, Belgrade

**Keywords:** reference intervals, indirect methods, thyroid parameters, referentni intervali, indirektne metode, parametri štitne žlezde

## Abstract

**Background:**

The aim of this study was to determine the reference intervals (RIs) for thyroid stimulating hormone (TSH), free thyroxine (FT4), free triiodothyronine (FT3) and FT3/FT4 ratio using indirect methods.

**Methods:**

We analyzed 1256 results TSH, FT4 and FT3 collected from a laboratory information system between 2017 and 2021. All measurements were performed on a Siemens ADVIA Centaur XP analyzer using the chemiluminescent immunoassay. We calculated the values of the 2.5th and 97.5th percentiles as recommended by the IFCC (CLSI C28-A3).

**Results:**

The RIs derived for TSH, FT4, FT3 and FT3/FT4 ratio were 0.34-4.10 mIU/L, 11.3-20.6 pmol/L, 3.5-6.32 pmol/L and 0.21-0.47, respectively. We found a significant difference between calculated RIs for the TSH and FT4 and those recommended by the manufacturer. Also, FT3 values were significantly higher in the group younger than 30 years relative to the fourth decade (5.26 vs. 5.02, p=0.005), the fifth decade (5.26 vs. 4.94, p=0.001), the sixth decade (5.26 vs. 4.87, p<0.001), the seventh decade (5.26 vs. 4.79, p<0.001) and the group older than 70 years old (5.26 vs. 4.55, p<0.001). Likewise, we found for TSH values and FT3/FT4 ratio a significant difference (p <0.001) between different age groups.

**Conclusions:**

The establishing RIs for the population of the Republic of Srpska were significantly differed from the recommended RIs by the manufacturer for TSH and FT4. Our results encourage other laboratories to develop their own RIs for thyroid parameters by applying CLSI recommendations.

## Abstract (sr)

**Uvod:**

Cilj ove studije bio je da se odrede referentni intervali (RI) tireotropnog hormona (TSH), slobodnog tiroksina (FT4), slobodnog trijodotironina (FT3) i odnosa FT3/FT4 indirektnom metodom procene referentnih intervala.

**Metode:**

Analizirali smo 1256 dobijenih vrednosti TSH, FT4 i FT3 u periodu između 2017. i 2021. godine. Rezultate smo uzeli iz laboratorijskog informacionog sistema. Sva merenja su izvedena na Siemens ADVIA Centaur XP analizatoru pomo}u hemiluminiscentnih imunohemijskih testova. Izračunali smo vrednosti 2,5-og i 97,5-og percentila prema preporuci IFCC-a (CLSI C28-A3).

**Rezultati:**

Procenjeni RI za TSH, FT4, FT3 i odnos FT3/FT4 bili su 0,34-4,10 mIU/L; 11,3-20,6 pmol/L; 3,5-6,32 pmol/L i 0,21-0,47. Utvrdili smo značajnu razliku između izračunatih RI za TSH i FT4 i onih koje preporučuje proizvođač. Takođe, vrednosti FT3 bile su značajno ve}e u grupi mlađoj od 30 godina u odnosu na četvrtu deceniju (5,26 vs. 5,02; p = 0,005), petu deceniju (5,26 vs. 4,94; p = 0,001), šestu deceniju (5,26 vs. 4,87; p<0,001), sedmu deceniju (5,26 vs. 4,79; p<0,001) i grupu stariju od 70 godina (5,26 vs. 4,55; p<0,001). Isto tako, za vrednosti TSH i odnos FT3/FT4 pronašli smo značajnu razliku (p <0,001) između različitih dobnih grupa.

**Zaključak:**

Procenjene vrednosti referentnih intervala za TSH i FT4 za stanovništvo Republike Srpske značajno su se razlikovale od preporučenih RI od strane proizvođača. Naši rezultati podstiču druge laboratorije da izrade sopstvene RI za parametre štitne žlezde primenom CLSI preporuka.

## Introduction

To make an appropriate diagnosis of thyroid disease and for more cost-effective monitoring of patients with altered thyroid function, it is necessary to ensure a quality and accurate laboratory analysis of thyroid function parameters: thyroid stimulating hormone (TSH), free thyroxine (FT4) and free triiodothyronine (FT3). TSH is the most sensitive marker for diagnosing subclinical functional thyroid disease. It is determined by the third generation methods with a sensitivity of 0.01 mIU/L [1]. However, standardization and harmonization of methods are still problematic and can lead to significant practical problems and have clinical consequences in the interpretation of laboratory findings [2] [3] [4] [5]. Furthermore, it has been shown that TSH reference intervals (RIs) should be redefined in different countries due to variability in regional iodine intake as well as used analytical methods [2].

For these reasons, it is necessary to make reference values for one's own population and not to use external sources, *i.e*., values proposed by the manufacturer. Using accurate RIs (median with 2.5th or 97.5th percentile) is imperative for laboratory professionals because comparing individual results with RIs is crucial for medical decisions. The validity of RI for serum TSH primarily affects hypothyroidism's diagnostic accuracy.

The direct method for a RI calculation is a chiefly recommended technique [6]. An alternative approach is the indirect method based on routinely collected patient samples used for diagnostic or monitoring purposes [7] [8].

**Figure 1:**
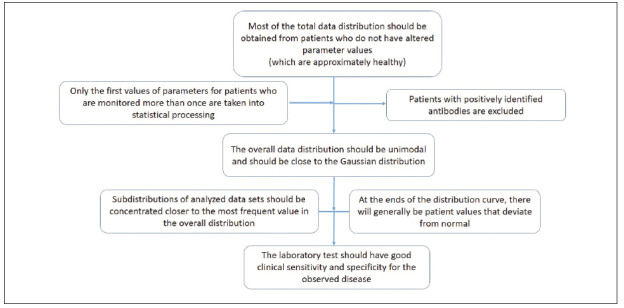
Proposed criteria for indirect method determination of RIs

Understanding the effects of within and between individual variability, analytical and preanalytical variability [3] [9], disease pathophysiology, and diagnosing the disease is crucial for both methods [10]. However, using the indirect approach in establishing RIs from patients' results is the simplest way to collect data and is significantly cheaper. Numerous studies explain the benefit of establishing indirect RLs for TSH, FT4 and FT3 from large databases stored in laboratory information systems [11] [12] [13] [14]. Also, RIs should be obtained in subjects whose thyroid dysfunction was ruled out based on biochemical filtration. For the establishment RI for TSH, TSH results should be excluded if FT3 and FT4 are outside the RI proposed by the manufacturer. This way of collecting data for RIs makes the reference population more similar to patients, including identical preanalytical conditions [15]. Laboratories are encouraged to use indirect methods to estimate RIs according to well-defined and recommended criteria by the International Clinical Federation Commission on Chemistry (IFCC) (CLSI C28-A3) [6] [8]. In Figure 1 we have presented the proposed criteria used in the indirect determination of RIs for thyroid parameters.

The goal of our study was to use indirect methods to estimate RIs for TSH, FT4, FT3 and FT3/FT4 ratio from results of the patients obtained during routine laboratory work. The investigation is conducted on the Republic of Srpska population.

## Materials and Methods

In this study, we analyzed the results of thyroid parameters (TSH, FT4, FT3) which have been collected from the laboratory information system (LIS) of the University Clinical Centre of the Republic of Srpska, Banja Luka. The measurements were performed on an ADVIA Centaur XP analyzer (Siemens Healthineers USA, United States) using the chemiluminescent immunoassay (CLIA). The collection period for the analyzed thyroid parameters was from October 1, 2017, to April 1, 2021.

The 1328 participants were enrolled in this study, older than 18 years, with predominantly female subjects (84%). The blood samples from outpatients were taken during the morning, between 7:00 and 11:00 a.m., at fasting. We excluded patients with positive antithyroid-peroxidase antibodies (>60 IU/mL) and antithyroglobulin antibodies (>4,1 IU/mL). Only the first result of each patient was included.

We evaluated patients' values within the RIs recommended by the manufacturer. Thus, when we estimate RIs for TSH, the FT4 and FT3 values should be within RIs but TSH values can be within, above or below RIs recommended by the manufacturer.

Quality control was performed using corresponding commercial control samples with low, medium, and high concentrations. The limit of quantitation (LoQ, functional sensitivity) of the ADVIA Centaur TSH3-Ultra assay was 0.008 mIU/L. Intra- and inter-assay coefficients of variation on the three levels of controls were for TSH 1.97%, 1.95%, 2.26% and 4.13%, 4.28%, 3.99%; for FT4 3.33%, 2.23%, 2.54% and 2.50%, 4.00%, 2.33%; for FT3 3.08%, 2.35%, 2.47% and 4.05%, 2.87%, 2.76%, respectively.

The estimated parameters were included in the external quality assessment scheme (Riqas, Randox). The RIs for TSH, FT4 and FT3 provided by the manufacturer were 0.55-4.78 mIU/L, 11.5-22.7 pmol/L and 3.5-6.5 pmol/L, respectively.

### Statistical analysis

Reference limits (RLs) were determined using statistical programmes MedCalc, version 12.1.4.0 (MedCalc Software, Belgium) and SPSS version 24.0 (SPSS Inc, USA). D'Agostino-Pearson test for normal distribution was used to test the distribution of the analyzed parameters. Suspected outliers were identified and omitted using the Tukey method [16] [17]. To estimate the indirect reference limits (RLs) for all the analyzed thyroid parameters non-parametric percentile method was used. Lower and upper limits, as 2.5^th^ and 97.5^th^ percentiles, were presented with 90% confidence interval (CI) for each limit. Considering Fraser's theory of »allowable bias« in laboratory tests, we tested whether the indirectly estimated RI significantly differs from the RI recommended by the manufacturer [18] [19]. We used a procedure proposed by Ozarda et al. [19] to normalize the RL differences. Firstly, we calculated the critical value for the upper RL differences (UL) and lower RL differences (LL). The numerator was equivalent to the upper limit (UL) ratio or lower limit (LL) ratio computed as a ratio of absolute differences in average UL (or LL) between indirectly estimated RLs and recommended RLs. The denominator corresponds to the standard deviation in calculating the RI, estimated as the average difference between UL and LL recommended by the manufacturer. To assess whether the calculated RIs differ from the recommended ones, we used the criterion of optimal analytical specification or desirable bias limit in laboratory tests as one-eighth (0.125) of the denominator. The RLs were considered divergent when the ratio exceeded the »optimal limits« for analytic bias (>0.125).

Additionally, thyroid parameters were analyzed according to decades of life and presented as box plots. To reveal the significance of differences between the subgroups relative to decades of life, the ANOVA test with a post-hoc Tukey test was performed.

## Results

**Figure 2:**
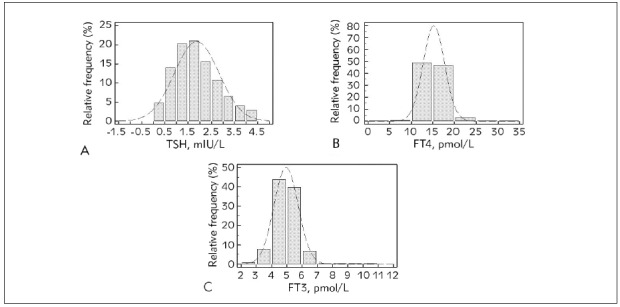
Distribution of the analyzed thyroid parameters: thyroid stimulating hormone, TSH; free thyroxine, FT4; free triiodothyronine FT3

In the Figure 2 we present the distribution of the analyzed thyroid parameters. All thyroid parameters show a skewed distribution with a long tail toward higher values. For further analyses, all values were log-transformed and we used Tukey's method for detecting outliers. After removing the outliers, the indirect reference values were determined in 1256 from 1328 data.

Calculated RLs for FT3/FT4 ratio were: 0.21 (0.20-0.22) for 2.5th percentile (90% CI) and 0.47 (0.46-0.48) for 97.5th percentile (90% CI), with median value (90% CI) of 0.33 (0.325-0.335). Reference interval width for indirectly calculated vs. recommended reference limits was 3.76 vs. 4.23, 9.3 vs. 11.2, and 2.9 vs. 3.0, for TSH, FT4 and FT3, respectively (Table 1). Further, we calculated critical values for UL and LL. Results were presented in Table 2. We found that there was a difference between the calculated and recommended ULs for TSH and FT4.

**Table 1:** Indirect estimation of RLs for the overall analyzed thyroid parameters determined on ADVIA Centaur XP Siemens immunochemistry analyzer Presents median, lower, and upper limits for all three analyzed thyroid parameters with corresponding 90 % CI

Analyzed thyroid parameters	2.5th percentile (90% CI)	50th percentile (90% CI)	97.5th percentile (90% CI)	Siemens manufacturer’s reference limits
TSH, mIU/L	0.34 (0.27–0.39)	1.73 (1.18–2.48)	4.10 (3.96–4.19)	0.55–4.78
FT4, pmol/L	11.3 (11.0–11.5)	15.01 (13.6–16.5)	20.6 (20.1–21.0)	11.5–22.7
FT3, pmol/L	3.5 (3.3–3.6)	4.9 (4.5–5.4)	6.4 (6.3–6.6)	3.5–6.5

**Table 2:** Comparison of the RLs calculated by indirect method with manufacturer recommended RLs RL, reference limit; LLi, lower reference limit calculated using the indirect method; LLr, lower reference limit recommended by the manufacturer; ULi, upper reference limit calculated using the indirect method; ULr, upper reference limit recommended by the manufacturer; ΔLL, critical lower limit ratio; ΔUL, critical upper limit ratio.

		TSH, mIU/L	FT4, pmol/L	FT3, pmol/L
Nominator	| LLi – LLr |	0.21	0.2	0
	| ULi – ULr |	0.68	2.1	0.1
Denominator	ULr – LLr	4.23	11.2	3
RL differences	LL	0.049	0.018	0
	UL	0.161	0.188	0.033

**Figure 3:**
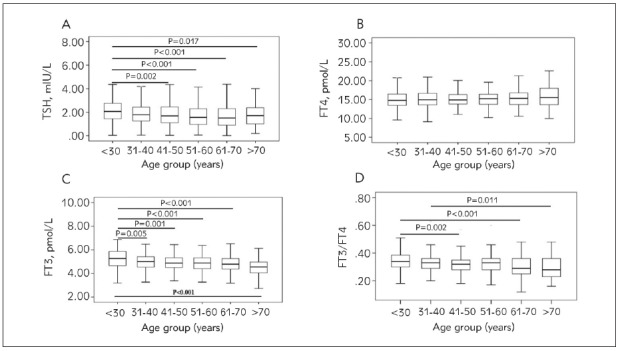
Median and interquartile range for the overall analyzed TSH, FT4, FT3 and FT3/FT4 values relative to decades of life

In the next step, we analyzed parameters according to age groups Figure 3. We have stratified groups as follows: younger than 30 years old (N=222), the fourth decade of life from 31 to 40 years old (N=320), the fifth decade of life from 41 to 50 years old (N=301), the sixth decade of life from 51 to 60 (N=164), the seventh decade of life from 61 to 70 (N=167) and older than 70 years old (N=82).

Differences in the reference values for the analyzed thyroid parameters relative to the decades of life were estimated using the Tukey HSD post hoc test, as set in one way analysis of variance (ANOVA). First, we found an overall significance value for the difference between groups for TSH (F (5.1251)= 6.147, p<0.001), FT3 (F (5.1251)=12.015, p<0.001) and FT3/FT4 ratio (F (= 5.276) = 6.147, p<0.001). A Tukey post hoc test revealed that the TSH values were statistically significantly higher in the group younger than 30 years relative to the fifth decade (2.06 vs. 1.69, p=0.002), the sixth decade (2.06 vs. 1.56, p<0.001), the seventh decade (2.06 vs. 1.51, p<0.001) and the group older than 70 years old (2.06 vs. 1.71, p=0.017). Additionally, FT3 values were statistically significantly higher in the group younger than 30 years relative to the fourth decade (5.26 vs. 5.02, p=0.005), the fifth decade (5.26 vs. 4.94, p=0.001), the sixth decade (5.26 vs. 4.87, p<0.001), the seventh decade (5.26 vs. 4.79, p<0.001) and the group older than 70 years old (5.26 vs. 4.55, p<0.001). FT3/FT4 index was statistically significantly higher in the group younger than 30 relative to the fifth decade (0.34 vs 0.32, p=0.002), to the seventh decade (0.34 vs 0.29, p<0.001) and relative to the group older than the 70 years old (0.34 vs. 0.30, p=0.011).

## Discussion

In this study, we established reference values for TSH, FT4 and FT3 in the population of the Republic of Srpska by indirect method *i.e.* using data stored in our information system. There was a statistically significant difference between calculated RIs for the TSH and FT4 and those recommended by the manufacturer. This indicates that it is necessary to define laboratory and method specific RLs for these thyroid parameters. The RIs for TSH in this study was apparently lower (0.34-4.1 mIU/L) than by the manufacturer (0.55-4.78 mIU/L). The RIs for TSH obtained on different populations, but the same analyzer (Siemens analyzer) show differences in the lower and upper limit of RIs in the ranges from 0.32 to 1.01 mIU/L and 3.00 to 5.51 mIU/L, respectively [20] [21] [22] [23] [24]. Therefore, our results are between these values but do not match them, which also favors establishing ours RIs. Also, this study's results agree with the general opinion that the upper TSH reference limits for outpatients should be below 4.5 mIU/L [25]. Nevertheless, laboratory guidelines show that more than 95% of healthy people have TSH below 2.5 mIU/L [26] which has not been confirmed in our study (Figure 2). Today, it seems to have the most published data on RIs on the Roche platform although it is necessary to publish RIs as many as possible for other platforms as well. The published data provide security to laboratory professionals in their daily, routine work. Our previous study showed that the TSH values obtained on Roche and Siemens analyzers well agree (the slope for the correlation of Roche and Siemens was 1.11 using the Passing-Bablok regression method) [2]. Also, in a similar study, we determined the TSH, FT4 and FT3 RIs for our population on a Roche analyzer [27]. We have noticed significant differences for TSH in the lower and upper limits (0.34 vs. 0.65 mIU/L and 4.1 vs. 5.39 mIU/L). This can be explained by the possible influence of environmental factors over the years, primarily the effect of iodine status. Research showed that in 2006 in the Republic of Srpska [28], there was not enough iodine in the diet, what could lead to such a high upper limit of TSH. The last study revealed a significantly lower value, indicating a significant improvement of the iodine status (unfortunately, there is no recent data on iodine in the diet in the Republic of Srpska). Also, the reason for this could be different methods for assessing RIs, the number of samples in the studies and the smaller number of men in the indirect method. The absence of a decline in serum FT4 values in our study further contributes to the evidence that there is adequate iodine intake in our population.

In addition, our results are more in line with the RIs population of the Republic of Serbia for TSH (0.35-4.10 vs. 0.42-3.67 mIU/L), if indirect method was used for determination of reference values [12].

According to ages, the shown changes for TSH are not clinically useful, which is in line with the results of other studies [29] [30]. Reasons for these changes may be due to physiological variables (e.g., menstrual cycle phase), individual variables, variables present in some non-thyroid diseases, iatrogenic factors such as thyroid and non-thyroid drugs, phlebotomy time, etc.

Surprisingly, both of our studies reported almost the same upper limit for FT4 and FT3 RIs (20.6 vs. 20.18 and 6.4 vs. 6.33 pmol/L), which to encourage us the future use of RIs obtained by indirect methods.

The best compliance of our RIs with the proposed values by the manufacturer was for FT3 which is, ultimately, crucial for a complete assessment of the success of the therapy. In addition, this cross-sectional study indicates that FT3 values change with ageing. Therefore, the existence of an age-related decrease in the circulating FT3 levels might represent a physiological mechanism already shown in some studies [31] [32].

The IFCC has so far made great efforts to standardize measurement for thyroid function tests, particularly for TSH, taking into account the different platforms used to measure these parameters. However uniform reference values for thyroid parameters have not yet been achieved. Therefore, routine clinical laboratories are advised to determine their own RIs following accepted consensus standards, such as those of the IFCC, National Academy of Clinical Biochemistry and CLSI [33] [34].

Additionally, we have examined RLs for FT3/FT4 ratio as useful parameter to detect thyroid disfunction [35] [36] [37] [38]. Some authors have pointed out that this ratio is positively correlated with TSH within the reference range of thyroid function in adults [36]. Our result of median value of FT3/FT4 ratio was in agreement with the parameter values examined by Chen and associates [35]. To our knowledge our study is the first that examined changes in the FT3/FT4 ratio by decades of age. Our results were also in agreement with Strich et al. [39] investigation. The authors have confirmed that TSH enhancement of FT4 to FT3 conversion is age dependent. These results indicate the importance of determining and monitoring free hormones ratio as an additional parameter that can help clinicians in assessing thyroid function. Also, more studies indicate a significant relationship between FT3/FT4 ratio and other diseases [39] [40]. The FT3/FT4 ratio would be useful in everyday practice.

This study has some limitations, primarily the small number of male respondents in the research and no recent data on sufficient iodine.

## Conclusion

The establishing and using your own thyroid hormone RIs provides a much better basis for diagnosing or considering treatment for thyroid dysfunction than using a manufacturer interval. The our study indicates the need for greater use of the FT3/FT4 ratio in routine work. In addition, these results should encourage more laboratories to apply CLSI recommendations in determining RIs for thyroid parameters, for their specific populations.

## Dodatak

### Acknowledgment

None.

### Conflict of interest statement

The authors reported no conflict of interest regarding the publication of this article.
